# Consensus recommendations for next-generation sequencing-based genetic testing in Rare Neurological diseases

**DOI:** 10.1038/s41431-026-02198-4

**Published:** 2026-07-28

**Authors:** Aleš Maver, Katja Lohmann, Lena-Marie Urbanczyk, Astri Arnesen, Ivo Barić, Peter Bauer, Kailash P. Bhatia, Sylvia Boesch, Fran Borovečki, Norbert Brüggemann, Zih-Hua Fang, Heinz Gabriel, Tobias B. Haack, Henry Houlden, Milena Janković, Erik-Jan Kamsteeg, Michelangelo Mancuso, Deborah Mascalzoni, Maria Judit Molnar, Alexander Münchau, Kornelia Neveling, Ivana Novaković, Borut Peterlin, Ludger Schols, Nika Schuermans, Katie Shiels, Marc Sturm, Rachel Taylor, Marina A. J. Tijssen, Lisenka E. L. M. Vissers, Victoria Williams, Holm Graessner

**Affiliations:** 1 Clinical Institute of Genomic Medicine, Ljubljana University Medical Centre, Ljubljana, Slovenia; 2 Institute of Neurogenetics, University of Luebeck, Luebeck, Germany; 3 Institute of Human Genetics, University of Luebeck, Luebeck, Germany; 4 Institute for Medical Genetics and Applied Genomics, University Hospital Tuebingen, Tuebingen, Germany; 5 European Federation of Neurological Associations, Brussels, Belgium; 6 Department of Pediatrics, University Hospital Center Zagreb, Kišpatićeva 12, Rebro, 10000 Zagreb, Croatia; 7 Centogene GmbH, Rostock, Germany; 8 Department of Clinical and Movement Neurosciences, UCL, Institute of Neurology, NHNN, London WC1N 3BG, UK Queen Square,; 9 Center for rare movement disorders Innsbruck, Dpt. of Neurology, Medical University Innsbruck, Innsbruck, Austria; 10 Department of Neurology, School of Medicine, University of Zagreb and University Hospital Center Zagreb, Zagreb, Croatia; 11 Section for Movement Disorders, Department of Neurology, University of Luebeck, Ratzeburger Allee 160, Luebeck, 23538 Germany; 12 German Center for Neurodegenerative Diseases, Tuebingen, Germany; 13 Center for Human Genetics, Paul-Ehrlich-Str. 23, D-72076 Tuebingen, Germany; 14 Center for Rare Diseases, University Hospital of Tuebingen, Tuebingen, Germany; 15 Department of Clinical Neurology, Institute of Neurology, London, UK; 16 Institute of Human Genetics, School of Medicine, University of Belgrade, Belgrade, Serbia; 17 Department of Human Genetics, Radboud University Medical Center, Nijmegen, the Netherlands; 18 Department of Clinical and Experimental Medicine, Neurological Institute, University of Pisa, Pisa, Italy; 19 Department of Public Health and Caring Sciences, Centre for Research Ethics and Bioethics, Uppsala University, Uppsala, Sweden; 20 Institute of Genomic Medicine and Rare Disorders, Semmelweis University, 1083 Budapest, Hungary; 21 Center for Rare Diseases, University of Lübeck, Lübeck, Germany; 22 Center of Neurology and Hertie Institute for Clinical Brain Research, University of Tübingen, Tübingen, Germany; 23 German Center for Neurodegenerative Diseases (DZNE), Tübingen, Germany; 24 Center for Medical Genetics, Ghent University Hospital, Ghent, Belgium; 25 EMQN CIC, ICE Building, 3 Exchange Quay, Salford, M5 3ED UK; 26 UMCG Expertise Centre movement disorders, Department of Neurology, University Medical Center Groningen (UMCG), Groningen, the Netherlands

**Keywords:** Genetic testing, Neurological disorders

## Abstract

We recently established an external quality assessment (EQA) scheme for next-generation sequencing (NGS) diagnostics in rare neurological disorders (RND) in collaboration with the EMQN. The first assessment rounds revealed limitations and variability in the quality and completeness of genetic testing reports. To improve and harmonize reporting in NGS-based diagnostics for RND, we identified 28 topics requiring recommendations based on EQA findings. These topics were grouped into four areas: clinical information, interpretation, methodology, and reporting. A team of 31 experts with relevant expertise was formed. Using an adapted Delphi approach, two rounds of surveys were conducted to prioritize the topics. Subsequently, area-specific expert groups met to formulate recommendations, followed by a final approval round using the five-finger consensus method involving all experts. In both Delphi rounds, all topics were rated as either very important (*n* = 12) or important (*n* = 16), and all were advanced for recommendation development. Consensus recommendations were achieved for 27 of the 28 topics. These include guidance on gene panel composition and updating, addressing disease-specific limitations of NGS (e.g., repeat expansion disorders), defining minimal quality parameters, and promoting sharing of variant interpretations. Each recommendation is supported by real-world examples. Due to differences in national healthcare frameworks and policies, consensus could not be reached regarding which patients should or should not undergo NGS testing. The dissemination of these recommendations is expected to improve the quality of genetic testing and reporting in RND diagnostics, promoting harmonization across laboratories and enabling easier comparison and interpretation of genetic testing reports.

## Introduction

Next-generation sequencing (NGS) technologies have become a foundational tool in the diagnosis of rare neurological diseases (RNDs), offering a rapid and cost-effective means to address their significant clinical and genetic heterogeneity [1–3]. As exome and genome sequencing increasingly serve as first-line diagnostic approaches, the reliability and reproducibility of NGS-based testing have become critical to ensuring equitable and accurate diagnoses for patients with RNDs [4, 5]. The complexity of NGS workflows—including library preparation, sequencing quality and depth, bioinformatic pipelines, variant interpretation, and clinical reporting—often results in substantial interlaboratory variability, which may result in disparate testing outcomes for patients across different institutions and countries [6, 7].

The European Reference Network for Rare Neurological Diseases (ERN-RND), established in 2017 [8], has prioritized improving access to high-quality genetic testing throughout the European Union (EU). As part of this mission, ERN-RND has implemented an external quality assessment (EQA) scheme for NGS diagnostics, which has been in routine annual operation since 2021 [7]. Notably, significant differences across laboratories performing genetic testing for RNDs were observed in all major areas, including methodological aspects, data interpretation, and reporting [7]. There is clearly a need for further harmonization by developing minimal standards for NGS-based diagnostic testing in RNDs.

While several broad guidelines for the major areas of NGS-based testing already exist [9–11], RNDs share several particularities that make them distinct from other genetic disorders. These include: (1) a relatively high diversity of mutational mechanisms, including single-nucleotide variants (SNVs), but also structural variants such as repeat expansions, (2) a high rate of genetic and phenotypic heterogeneity [12], as well as, (3) specific clinical context considerations, e.g., for (predictive) genetic testing in adult–onset neurodegenerative disorders. Furthermore, the available guidelines for NGS testing are often based on the participation of experts in the field of molecular testing, whereas we anticipate that the involvement of stakeholders from more diverse backgrounds could assist in developing guidelines that are better adapted to the practical context of genetic testing.

To address these gaps, we developed comprehensive recommendations to standardize the processes of NGS-based diagnostics in RND, based on the participation of experts from diverse fields. The aim of this work was to provide clinically oriented practice recommendations for laboratories, clinical geneticists and neurologists to support consistent, high-quality NGS testing, interpretation and reporting in RND diagnostics across institutions and countries.

## Methods

**Fig. 1:**
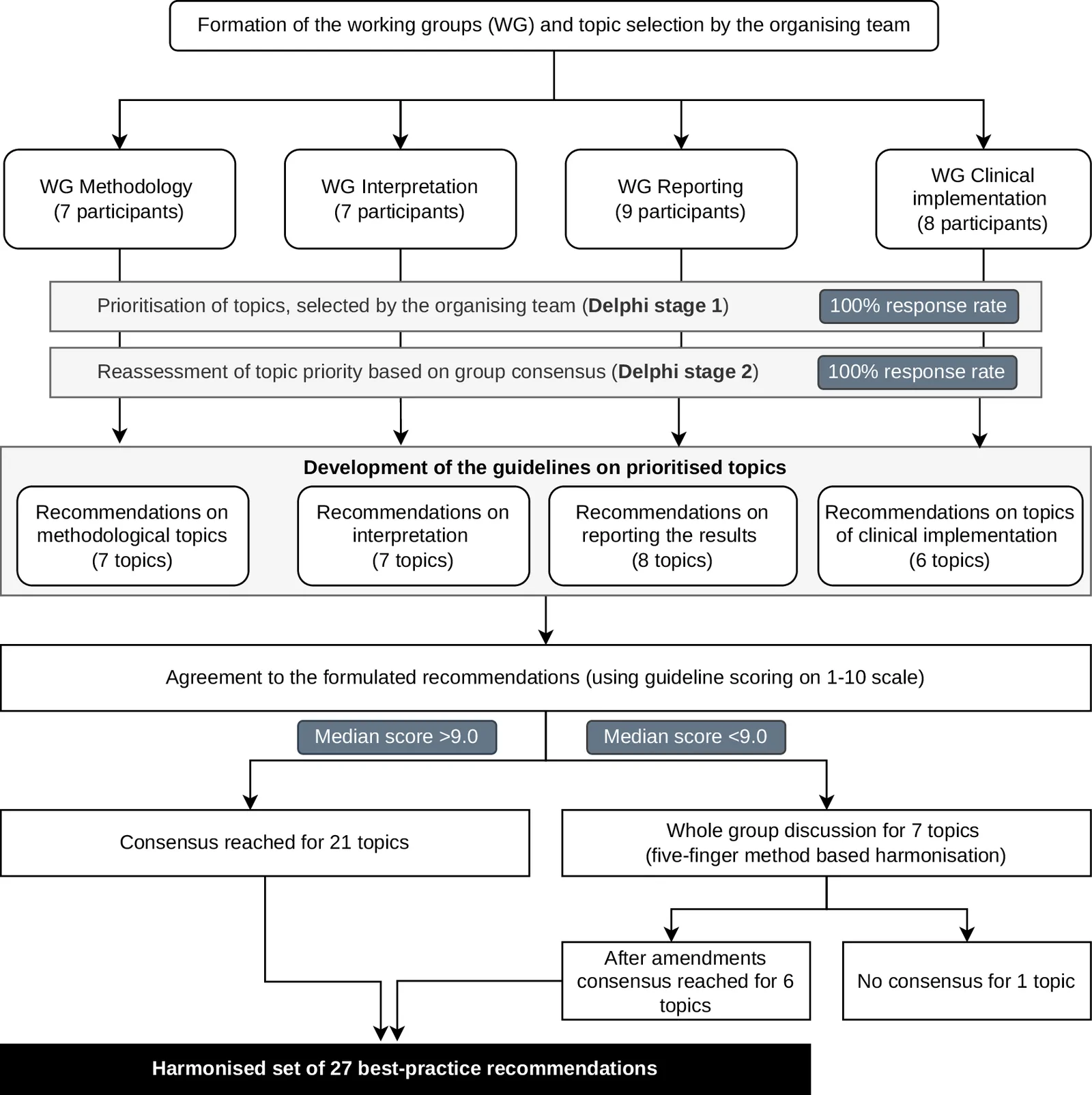
The process undertaken to develop best practice recommendations for NGS-based testing in RNDs.

The process of development of best practice recommendations is presented in Fig. 1 and detailed in the sections below.

### Identification of topics for recommendation development

We based the topic selection for the recommendation development on the outcomes and experience from the EQA scheme for diagnostic NGS in RNDs, which has taken place annually since 2021 [7]. During this process, 28 areas of need for clear recommendations were identified (Table 1) and ultimately, for 27 topics a consensus could be reached (see Results section).

**Table 1:** The key points of recommendations for NGS testing in RNDs.

Category	ID	Consensus stage^a^	Topic	Key statements^b^	Practical illustration (applicability in routine RND diagnostics)^b^
**Clinical**	**R1**	S	**Definition of a minimal common gene content (panel) for genetic testing of RNDs**	Clearly define analyzed genes and variant types. Use regularly updated, disease-specific dynamic gene panels (e.g. PanelApp). Avoid outdated or static gene lists. Prioritize genes with strong clinical validity.	The experience with the ERN-RND scheme has shown that clinically relevant variants may be missed if in-house panels are not regularly updated [7]. The use of solutions like PanelApp can ensure current gene content, thereby minimizing missed diagnoses.
**Interpretation**	**R2**	S	**Recommendation on updating the gene panel content**	Review gene panels every 3–6 months; annually at minimum. Promptly integrate newly validated genes. Use version control to track updates. Exclude genes with insufficient evidence.	A patient with Parkinson’s disease (PD) was tested in 2025 with a 2023 gene panel, which resulted in a missed *RAB32* variant (OMIM: 620923 added as PD gene in 2024), leading to an undetected pathogenic variant inherited by two offspring [17, 18]. Routine updating of panels is therefore needed to avoid missed diagnoses.
**Methodology**	**R3**	S	**Handling NGS-related limitations for the genes included in the analysed gene panel**	State limitations in detecting specific variant classes. Identify gaps in assay capability (e.g., STRs, deep intronic). Recommend complementary assays when needed.	A patient with chorea tested by exome sequencing returned negative because expansion detection was not performed; re-testing a year later identified a Huntington disease-related expansion (OMIM: 143100). Clear guidance on potentially missed variant types and follow-up testing would have prevented a diagnostic delay.
**Reporting**	**R4**	S	**The recommendation on the use of tests complementary to NGS-based testing**	Suggest follow-up testing for undetectable pathogenic variant classes. Provide phenotype-driven recommendations (e.g., RP-PCR, *FMR1* sizing).	A 59-year-old patient with suspected cerebellar ataxia, neuropathy, and vestibular areflexia syndrome (CANVAS; OMIM: 614575) had a negative exome sequencing result; *RFC1* expansion was not reliably assessed and the report lacked clear follow-up guidance. Clear instructions in the primary conclusions are necessary to ensure appropriate follow-up testing.
**Methodology**	**R5**	D	**Recommendations on the minimal set of bioinformatic analyses that should be performed**	Apply core bioinformatics: QC, alignment, variant calling/filtering. Include post-calling quality metrics to ensure data reliability.	A spastic paraplegia (SPG) patient tested by WGS had only a single heterozygous pathogenic *SPG7* variant detected (*SPG7* is linked to an autosomal recessive form of SPG, OMIM: 607259); low genome sequencing coverage missed another pathogenic variant in Exon 1. Clear reporting of experiment-specific quality metrics is therefore essential to guide follow-up testing.
**Methodology**	**R6**	S	**The use of extended bioinformatic analyses in the analysis of NGS data**	Detect and report SNVs, indels, CNVs, SVs, STRs when clinically relevant. Explicitly state covered variant classes. Use validated methods and confirm important findings.	An early-onset Parkinson’s disease (PD) patient was investigated using exome sequencing, revealing only a single heterozygous *PRKN* loss-of-function SNV (*PRKN* is linked to an autosomal recessive form of PD, OMIM: 600116). The laboratory’s CNV-calling algorithm was not validated for single exon deletions/duplications, leaving the patient undiagnosed. Reports should clearly indicate such limitations and provide guidance for follow-up testing, including information on CNV detection sensitivity, highlighted in the main findings of the report.
**Methodology**	**R7**	S	**Provision of data on the bioinformatic algorithms and pipelines used**	Report tools, software versions, and parameters used. Specify genome build and annotation sources. Ensure reproducibility and transparency.	A patient with suspected mitochondrial encephalomyopathy, lactic acidosis, and stroke-like episodes (MELAS; OMIM: 540000) was initially tested using a pipeline filtering out variants with an allelic ratio below 10%, resulting in a negative report. Subsequent testing of another tissue revealed a recurrent pathogenic variant in the mitochondrial DNA, which had been present in the original data but filtered out. Clear reporting of analytical steps (in this case, declaration if mitochondrial variants were analysed and if low-heteroplasmy variants were sought for) is essential to avoid missed diagnoses.
**Methodology**	**R8**	S	**Recommendation for strategies and requirements for NGS pipeline validation**	Validate with high-confidence datasets (e.g. GIAB). Report sensitivity, specificity, and reproducibility. Demonstrate validation for all relevant variant types.	A patient with suspected dystonia was tested by exome sequencing, but poor detection of indels in the variant analysis step led to a missed diagnosis. Routine pipeline validation could assist the laboratory in identifying and remediating such issues early and preventing missed diagnoses.
**Methodology**	**R9**	S	**Minimal quality parameters that need to be assessed in the sequencing experiment**	Report the mean and minimum coverage. Include % bases meeting minimum threshold (e.g. ≥20×). Define targeted regions (e.g,. exons ±20 bp).	Coverage thresholds vary widely between laboratories, affecting variant detection. Genome sequencing at 10x may miss variants detectable at 20x, and even exomes with high median coverage (e.g., >100x) can have poorly covered critical regions. Consistent reporting of QC metrics, including percentage of regions with insufficient coverage, is essential. Some regions remain difficult to analyse with short-read NGS [19].
**Reporting**	**R10**	S	**Minimal quality and test parameters that need to be included in the report**	Include platform, enrichment method, read length, and coverage metrics. Provide sufficient technical detail for reinterpretation.	Re-evaluation of a dystonia case after a negative 2013 exome revealed non-homogeneous coverage, with >5% of regions below 10x. Repeated exome sequencing identified a pathogenic variant in the first exon of *GCH1* (which was poorly covered in the initial exome test), establishing the diagnosis of dopa-responsive dystonia (OMIM: 128230) and enabling timely interventions. Minimal quality criteria should be clearly stated to guide clinicians and support potential reanalysis or further testing.
**Methodology**	**R11**	D	**Management of the gaps in sequencing data**	Highlight regions with inadequate coverage. Indicate impact on diagnostic confidence. Recommend reanalysis or further testing as needed.	A child with suspected hypomyelinating leukodystrophy received a negative report; a frequent promoter variant in *UFM1* (OMIM: 617899) was missed due to non-capture of the *UFM1* promoter region by panel sequencing. The laboratory listed *UFM1* in the panel, giving the impression it had been fully assessed. Laboratories should indicate potential sequencing gaps and recommend follow-up testing to avoid false negatives.
**Clinical**	**R12**	S	**Define broad disease-specific recommendations for NGS-based testing in RNDs**	Justify the selection of panel/WES/WGS based on phenotype and utility. Explain coverage and variant detection limitations. Recommend additional tests if indicated.	A patient with unspecified ataxia may benefit from early whole genome sequencing to detect small variants and CNVs, but short-read NGS will miss complex structural variants, including *RFC1* repeat expansions causing CANVAS (OMIM: 614575). Laboratories should consider the limitations of NGS for expansion disorders, particularly when CANVAS is suspected.
**Reporting & Clinical**	**R13**	S	**Address the disease-specific limitations of NGS-based testing**	Describe detection limits for all variant types. State assay’s analytical constraints in reports. Link limitations to clinical recommendations.	NGS-based testing shows high sensitivity for expansion disorders (97.3% [20]), but is insufficient when there is a high a priori probability for a specific expansion (e.g., Huntington disease OMIM: 143100). Short-read NGS may not accurately determine repeat sizes, which are critical for prognosis and reproductive decisions.
**Interpretation**	**R14**	S	**Disease-specific variant interpretation guidelines**	Interpret repeat expansions based on repeat number, interruptions, and locus-specific rules. Use consensus guidelines and up-to-date literature. Consider mosaicism and clinical correlation.	A mother with a premutation *FMR1* allele (77 CGG) had a son with 103 CGG and a daughter with 220–1822 CGG. The son developed Fragile X-associated tremor/ataxia syndrome (FXTAS; OMIM: 300623) in later adulthood, while the daughter had fragile X syndrome (OMIM: 300624). This example illustrates the importance of laboratories to consider findings in a disease-specific context (here, noting that different expansions can lead to distinct phenotypes) [21].
**Interpretation**	**R15**	D	**Recommendations on the use of variant interpretation guidelines**	Use universal ACMG guidelines for variant classification In-house systems are acceptable if explained and follow ACMG principles Support and build expert panels (e.g., ClinGen VCEPs) Share outcomes via reviews and databases like ClinVar Do not rely only on ClinVar, esp. low confidence (1-2 star) entries Be cautious with automated tools (which may overinterpret e.g., truncating variants) Expert knowledge and clinical interpretation essential	Heterozygous pathogenic variants in *LRRK2* are a relatively frequent cause of monogenic Parkinson’s disease (PD, OMIM: 607060). Disease mechanism appears to involve increased LRRK2 kinase activity from missense variants, while heterozygous truncating variants have not been linked to PD [22]. Automated or unsupervised pathogenicity scoring may misclassify a previously reported truncating variant (c.6187_6191del) as likely pathogenic due to incorrect application of the PVS1 criterion [23].
**Interpretation**	**R16**	S	**Systematic reporting of evidence used to classify identified variants**	Mention ACMG criteria used, but prefer plain language Provide info on variant frequency, in-silico predictions, conservation, functional tests, segregation, zygosity Watch out for pitfalls: population-specific frequencies, prediction limits, test variety Indicate if the variant was previously published, and provide citations List all reports, if only a few are available; for well-known variants, cite original + recent review	Variant location is critical when multiple protein isoforms exist. In hereditary spastic paraplegia (HSP), heterozygous pathogenic *SPAST* variants affect two isoforms, M1- and M87-spastin (OMIM: 182601). Disease involves both haploinsufficiency and gain-of-toxicity mechanisms, with variants affecting only M1 linked to a milder phenotype [24].
**Interpretation**	**R17**	S	**Sharing variant classifications**	Share variant interpretations within diagnostic and research communities Include sharing in your internal workflow Especially important for VUS to gather evidence and update classification Provide phenotypic information, if possible	Numerous cases exist where a VUS was upgraded to pathogenic through collaborative efforts. A patient with early-onset, levodopa-responsive tremor–parkinsonism carried compound heterozygous *WARS2* variants in 2018, including a missense VUS. Additional reports expanded the phenotype from predominantly neurodevelopmental (OMIM: 617710) to include prominent movement disorders such as tremor-parkinsonism (OMIM: 619738). Accumulated evidence and functional tests established the variant as pathogenic [25]. Exchanging VUS data and collecting cumulative evidence can guide variant interpretation.
**Interpretation**	**R18**	S	**The use of match-making networks to improve the outcomes of patients through data-sharing**	Use match-making networks alongside ClinVar to improve variant interpretation and establish gene-disease validity (particularly for novel or recently reported genes) Especially helpful for VUS and potentially actionable conditions Guided by resources and ethical issues like patient consent	Match-making tools like GeneMatcher are widely used in research, particularly for neurodevelopmental disorders, to validate disease-causing variants. In rare neurological diseases, match-making helped establish *VPS13D* as a novel gene for spastic ataxia [26].
**Reporting**	**R19**	D	**Format and content of the NGS-testing reports**	Follow a clear, structured, standardized format for clarity and conciseness Encourage uniform structure (national level), align with ESHG [27–29] and ISO 15189 Highlight key findings, prevent misinterpretation Standardized format improves communication and comparability Allow some flexibility to fit different styles/workflows	A clinician received a 7-page NGS report with the principal finding on page 3 and multiple VUS on page 4. Misinterpreting a VUS as the key finding led to an incorrect diagnosis and inappropriate patient management. Reports should be concise, clearly highlighting the principal finding early, and structured to minimize confusion from additional information.
**Reporting**	**R20**	S	**The use of standard reporting language to minimize ambiguity**	Use standardized, well-defined terminology for clarity and consistency Link findings to referral indication Transparently report uncertain results and follow-up guidance Follow international guidelines (ACMG, HGVS) Maintain flexibility for specific clinical contexts Support cross-border collaboration	A 71-year-old patient with frontotemporal dementia underwent whole genome sequencing, and a VUS in *MAPT* (OMIM: 600274) was reported as a potential cause without clearly stating the uncertainty. Relatives requested pre-symptomatic testing based on this report. Standardized reporting, aligned with best-practice guidelines (e.g., ACGS), reduces misinterpretation and ensures consistency across institutions.
**Reporting**	**R21**	S	**Recommendations on the variants to report**	Clearly define which variants to include (prioritize clinically relevant) Report ‘hot’ VUS only if emerging evidence supports pathogenicity Avoid unnecessary or confusing findings Follow international guidelines with disease-specific flexibility Exclude clinically irrelevant results (e.g., high-frequency recessive carrier status) Transparent communication with clinicians and patients is key	Existing recommendations for genome sequencing reporting advise limiting reports to (likely) pathogenic variants and VUS that are clinically consistent with the referral and can be further clarified [11], supporting the current recommendation.
**Clinical**	**R22**	S	**Define the minimal set of clinical information and guidance to be included in the report**	Reports should be standalone with essential clinical guidance Always recommend genetic counseling when a diagnosis is made Advise further testing if needed (e.g., segregation analysis) Indicate treatment options and refer to expert networks (ERNs) Management advice should align with the latest clinical guidelines Standardization improves clarity, communication and patient care	When a patient and clinician receive a genetic testing report, determining its relevance for management can be challenging. For *GCH1*-associated dopa-responsive dystonia (OMIM: 128230), the laboratory should indicate the need for follow-up sequencing in parental samples, note that penetrance depends on gender, and highlight available treatment options [30].
**Reporting**	**R23**	D	**Recommendations to adjust complexity and information in the report to the target audience (the recipient)**	Provide a concise summary of key findings upfront for quick understanding Structure reports for geneticists and non-genetic clinicians with brief explanations Balance readability and scientific accuracy, avoid oversimplifying Reports should be usable standalone across institutions Detailed technical data should be stored separately Keep reports patient/clinician-focused	Laboratories should include essential guidance on mode of inheritance, recurrence risk, and available or imminent therapeutic interventions that may impact patient management.
**Interpretation**	**R24**	S	**Reinterpretation of the NGS data**	Reinterpretation can lead to new diagnoses by integrating new evidence Two layers: gene level (new disease genes) and variant level (updated interpretations) Important for VUS; aim for re-interpretation every 2 years if feasible AI-based pipelines may help future reinterpretation Update reports if classification changes clinical interpretation Indicate that variant interpretation is a “snapshot” and can change, particularly in the case of VUS findings Consider patient consent challenges	Systematic re-analysis by the Solve-RD consortium provided a molecular diagnosis in 12.6% of over 6000 patients, highlighting the value of re-analysis [31].
**Reporting**	**R25**	S	**Unsolicited findings in NGS-based testing**	Report unsolicited findings only if the patient explicitly consents during pre-test counseling Report only clinically relevant/actionable findings, exclude VUS to minimize patient anxiety Use virtual panels to limit unwanted findings Ensure proper genetic counseling pre- and post-test Follow (inter)national regulations Standardize protocols across labs for managing unsolicited findings	Patients and professionals should be informed that genetic testing can identify unsolicited findings unrelated to the referral diagnosis. NGS-based approaches require proper consent, and healthcare systems must ensure follow-up counseling and management [32].
**Reporting**	**R26**	S	**Reporting of variants of uncertain significance**	Define clear criteria for reporting VUS, limit it to relevant clinical cases or strong disease links Follow country-specific regulations and state VUS reporting policies Context-dependent reporting balancing benefits and risks Avoid unnecessary VUS reporting to prevent confusion and anxiety Recommend genetic counseling when reporting VUS Monitor and reclassify VUS over time; inform clinicians and patients Optionally report VUS separately only if patient consents	A patient with hereditary spastic paraplegia initially received a negative exome report. Years later, reinterpretation revealed a likely pathogenic *SPAST* variant (OMIM: 182601) affecting intronic splicing. Segregation studies could have enabled earlier reclassification, but the laboratory policy excluded reporting VUS. Laboratories should clearly state their VUS reporting policies and indicate when follow-up testing could lead to reclassification [33].
**Clinical**	**R27**	D	**Recommendations for reporting carrier status**	Report carrier status only if relevant to the clinical indication Include heterozygous variants in recessive conditions when the second variant is missing and the phenotype fits or the referral phenotype is highly specific For carrier status as an unsolicited finding, follow patient consent and report only the recommended genes Avoid reporting unrelated carrier status except for reproductive counseling Ethical and informed consent considerations are vital Recommend genetic counseling to guide patients	A 4-year-old with subtle early ataxia underwent exome sequencing, including CNV and repeat expansion analyses, revealing only a single heterozygous pathogenic variant in the *NPC1*, associated with Niemann–Pick disease type C (OMIM: 257220), an autosomal recessive neurodegenerative disorder. Follow-up Sanger sequencing identified a second splice-region variant missed by NGS due to a homopolymer stretch [32]. Miglustat therapy was initiated [33].

^a^ S – Consensus reached based on the high consensus score of the statement; D – Consensus reached after additional discussion and/or amendments required before final harmonization.

^b^ Detailed clarification of each recommendation along with extended presentation of clinical applicability examples are provided in the Supplementary Information text.

*ACMG* American College of Medical Genetics and Genomics, *CANVAS* cerebellar ataxia, neuropathy, and vestibular areflexia syndrome, *CNV* copy number variant, *FXTAS* fragile X-associated tremor/ataxia syndrome, *HSP* hereditary spastic paraplegia, *MELAS* mitochondrial encephalomyopathy, lactic acidosis, and stroke-like episodes, *mtDNA* mitochondrial DNA, *NGS* next-generation sequencing, *OMIM* Online Mendelian Inheritance in Man, *PD* Parkinson’s disease, *QC* quality control, *SNV* single nucleotide variant, *STR* short tandem repeat, *VUS* variant of uncertain significance, *WGS* whole genome sequencing.

### Formation of the working group

The working group (WG) was assembled to ensure a diverse representation of stakeholders participating in the process of genetic diagnostics of RNDs (Supplementary Table 1). Experts participating in the consensus process were selected from the European Reference Network for Rare Neurological Diseases (ERN-RND), a network of nationally designated centres of expertise for RNDs across Europe. Participants were selected to ensure representation of all key areas of expertise relevant to RND diagnostics and care, including clinicians, laboratory specialists, researchers, patient organisations, academia, industry, professional societies, EMQN, and ERN-RND. The composition of the group was further designed to achieve broad geographical representation across Europe and comprehensive coverage of the main stakeholder groups involved in NGS-based diagnostics. Selection aimed to ensure broad geographical and institutional representation in order to reflect differences in institutional practices and national legal frameworks relevant to genetic testing and reporting. The final expert panel represented 11 European countries. Potential conflicts of interest were considered during the expert selection process and during development of the recommendations. Experts participating in the consensus process were formally involved throughout the guideline development process and were specifically contacted during each Delphi round to encourage continued participation and minimize participant attrition. As a result, attrition between rounds was limited and did not substantially affect the overall geographical, institutional or disciplinary representation of the expert panel.

Stakeholders engaged in the recommendation development process included members of the EQA organizing and performing team (*n* = 3), specialists in neurology and clinical genetics (*n* = 7), representatives of patient advocacy groups (*n* = 1), legal experts (*n* = 1), professionals in quality assurance (*n* = 3), delegates from relevant professional bodies (*n* = 5), experts in sequencing and data analysis (*n* = 3), and experts in clinical variant interpretation (*n* = 8). The WG members were formally invited and were included in the WG following their acceptance. They were assigned to four focused WGs based on their area of expertise. Seven to nine members of each group focused on: Methodology (*n* = 7), Interpretation (*n* = 7), Reporting (*n* = 9), or Clinical Implementation (*n* = 8) (Supplementary Table 1).

### Prioritization of the relevant topics

In a first step, all WG members were asked to assign importance to each topic selected by the organizing team. Two Delphi rounds were undertaken to achieve this. The first Delphi round was initiated in May 2023, when the WG members were asked to prioritize the broad areas of recommendations and vote on the importance of selected topics. All WG members were asked to rank the importance of topics on a 5-point scale (1 =very unimportant to 5 = very important). The panelists’ responses were then used to calculate the levels of importance. An average importance score was assigned to each topic. During this stage, we also asked participants to provide additional comments for topic formulation.

The second Delphi round was conducted in February 2024, allowing all WG participants to reassess the topic’s importance based on the results of the first Delphi round. For this, the panelists were informed about the median importance score of all topics and their own previous rating. Only the panelists who responded to the online questionnaire in the first Delphi round (*n*  =  27) were asked to respond to the ranked topics in the second Delphi round. An identical 5-point scale was used to re-score topics.

Complete responses from all participants were obtained at each stage of the Delphi process.

The topic was considered sufficiently important if the mean score of a topic exceeded an average value of 3.5 and if the majority of the votes rated the topic as “important” or “very important”.

To facilitate the process of drafting recommendations, the 28 topics selected during the survey phase were assigned to four thematic categories: Methodology (*n* = 7), Interpretation (*n* = 7), Reporting (*n* = 8), and Clinical Implementation (*n* = 6). These categories reflect the major aspects of the diagnostic NGS process.

### Development of the best practice recommendations

The recommendations were initially formulated by each focused WG. In November 2024, the coordinating member of each focused WG defined the strategy to formulate recommendations, either in an initial WG meeting or by forming a draft version of recommendations, which was provided to focused WG members for rating and comments.

After draft recommendations were prepared by the focused WGs, they were disseminated to all WGs using an online questionnaire, where all members could vote on their agreement with each statement and provide optional comments on their vote. All members of WG could rate the statements with a score ranging from 1 to 10 (where score 1 indicated complete disagreement and score 10 indicated complete agreement). The ratings obtained in this manner were used to select statements for consensus discussions in the final WG meeting.

For the final recommendation harmonization, the topics rated with an average score below 9.0 were selected for further discussion. These topics were presented at the final consensus meeting, held in May 2025, where participants could discuss their opinions and propose amendments to the statements. The “five-finger” consensus method was employed to facilitate participatory decision-making for reaching a consensus on the recommendations [13]. Participants indicated their level of agreement with a proposal by voting on statements with a score of one to five (“fingers”), where a score of 5 indicated full agreement and a score of 1 indicated full disagreement [14]. A consensus was reached when all WG participants voted with a score of 3 (“will go with the group’s decision”) or more on the statement. When a participant expressed disagreement with a score of 1 or 2, they were asked to propose modifications to the statement, after which another voting round was conducted until a full consensus was reached. WG members who were unable to participate in the consensus meeting were followed up by email using the same procedure. If no consensus could be reached, the topic was left without a recommendation.

Mapping between Delphi-prioritized topics and the final recommendations is presented in Table 1, in which each topic directly corresponds to the associated recommendation identifier and thematic working group.

## Results

Altogether, 31 members representing diverse aspects of genetic testing were included in the WG to develop best practice recommendations for NGS-based testing in RND. The majority of recommendations were developed within focused WGs consisting of seven to nine members with expertise in (at least) one of 4 principal fields: NGS methodology, variant interpretation, diagnostic reporting, and the clinical use of NGS-testing results.

### Selection of areas of importance in NGS testing of RNDs

Initially, potential topics for the development of recommendations were selected based on the weaknesses observed through the EQA scheme within the ERN-RND network. Subsequently, WG members were asked to prioritize the topics where the need for harmonization is most pronounced. The results of the two-tiered Delphi survey indicated that the majority of WG members viewed all issues recognized during provision of the EQA scheme as important (*n* = 16) or very important (*n* = 12). The topics and the results of the Delphi second round are presented in Table 1.

### Best practice recommendations for NGS in genetic diagnostics of RNDs

Altogether, 28 recommendation statements were formulated by four focused WGs, addressing the fields of NGS methodology, variant interpretation, reporting and clinical implementation. These were delivered to the full WG to reach consensus. The initial voting round revealed a high rate of consensus for 21 statements, reaching an average score above 9.0. The remaining 7 statements were further discussed in an online WG consensus meeting or by email with members who were unable to participate in the meeting, using the 5-finger method to measure and reach consensus. Modifications were included in six of these recommendations, and consensus was reached (all WG members voted at least with 3). Notably, for the criterion on “The clinical context of NGS-based testing” no consensus was reached. This topic was rated high, i.e., “very important” in Delphi, however, the discussion revealed that there were insurmountable discrepancies in the idea to restrict NGS-based testing to preselected patients, for instance, based on phenotypic features or disease onset. Therefore, the summary of the recommendations in Table 1 (key points) and Supplementary Information (full recommendations with examples) lists only 27 recommendations.

## Discussion

To our knowledge, this work presents the first set of expert recommendations specifically developed to improve and harmonize NGS-based diagnostics for RNDs. They address major components of the genetic testing process—including methodology, interpretation, reporting, and clinical implementation—and are the result of an interdisciplinary participation of diverse stakeholders involved in genetic testing of RNDs.

By engaging 31 professionals from diverse domains—clinical genetics, neurology, bioinformatics, laboratory diagnostics, quality assurance, and patient advocacy—we ensured that the recommendations reflect the complex and multifaceted nature of genetic testing for RNDs. This diversity of expertise helped align technical standards with clinical utility, laboratory feasibility, ethical frameworks, and patient-centered care. Importantly, the process also included participants affiliated with multiple professional organizations and societies, including the European Academy of Neurology, the EuroGentest committee at the European Society of Human Genetics, the Movement Disorders Society and the EMQN, facilitating broader alignment with existing European and international standards as well as - by endorsement - supporting the take-up of the recommendations by genetic diagnostic laboratories in Europe and globally.

The recommendations were developed through a robust and well-established process that facilitated consensus among a diverse set of experts from 11 European countries. The process began by leveraging insights from ERN-RND EQA scheme [7]—an initiative that has revealed significant variability in current diagnostic practices. Based on this experience, we identified 28 relevant topics that require guidance and harmonization. We applied a two-tiered Delphi approach to prioritize these topics, ensuring that all recommendations address areas identified as important by the diverse group of experts. The recommendation statements were developed through focused WGs, and consensus was achieved using the five-finger consensus method, which allowed for participatory decision-making and ensured agreement across all members of the WG. A consensus could be reached for all items but one recommendation, as there was no approval on the question of whom to test (clinical context). While some participants felt it would be important to restrict NGS-based testing to patients with a high likelihood of a monogenic cause, e.g., early-onset, familial, syndromic, or complex/atypical neurological presentations, and avoid testing sporadic, late-onset, or more common phenotypes without an established clinical indication of a genetic disorder, others favored to omit any limitations here and allow NGS-based testing as the first tier genetic test, and to leave economic circumstances and available resources guide the selection of patients to be NGS-tested [15, 16].

We anticipate the immediate applicability of the developed guidelines and will monitor their use in future rounds of NGS-based EQA schemes in ERN-RND. As such, they will serve as both a framework for evaluating current practice and a set of measurable targets for improvement. This immediate feedback loop between recommendations and implementation provides an opportunity not only to assess but also to actively improve the quality of diagnostic services across and beyond the ERN-RND network. As a matter of fact, this aligns with the principal modus operandi of ERNs: basing the development of a healthcare intervention - i.e. the recommendations - on an analysis of the heterogeneity and inequality of a healthcare service provision in Europe - i.e., diagnostic NGS testing of RND patients - as well as using the measurable target - i.e., the ERN-RND NGS EQA - for both a measurement of the effect of an intervention and to improve the intervention as such. Moreover, we envision the recommendations as a foundation for harmonized genetic reporting practices across Europe, promoting diagnostic consistency, clinical equity, and cross-border interoperability. The European Academy of Neurology has already endorsed the present document.

The recommendations also address specific needs of the RND field that are not sufficiently covered by existing general NGS guidelines. For example, they account for the frequent presence of complex mutational mechanisms, such as repeat expansions, the high degree of genetic heterogeneity, and the clinical challenges associated with testing in adult-onset neurological conditions. Furthermore, they emphasize updating gene panel content, reporting disease-specific technical limitations, ensuring the attainment of minimum quality parameters for sequencing, and encouraging the sharing of variant interpretations. To promote the applicability of the guidelines and clarify the rationale for their inclusion, we also provide real-world examples to support their implementation in clinical laboratories (Table 1 and Supplementary Information).

Although we employed a rigorous guideline preparation procedure, there are limitations that should be considered. First, despite efforts to ensure broad geographical and multidisciplinary representation, some degree of selection bias may have been introduced through the inclusion of experts primarily affiliated with ERN-RND-associated centres and collaborating institutions. Second, the composition of the expert panel may have influenced the prioritization and interpretation of recommendations, reflecting the clinical, technical and regulatory perspectives represented within the group. Finally, as with all Delphi-based consensus approaches, the recommendations are influenced by expert opinion and consensus methodology, which may not fully capture all regional practices, institutional workflows or emerging evidence.

In summary, this work represents a comprehensive, consensus-driven, and implementable effort to establish a quality-controlled standard of genetic diagnostics in RNDs. Through direct integration into the RND NGS EQA scheme and alignment with stakeholder priorities, these recommendations provide a blueprint for both measurable improvement and long-term harmonization in NGS-based diagnostics across Europe and beyond, thereby contributing to improved patient care.

## Supplementary information

Supplementary Tables

Supplementary information - The best practice recommendations

## Data Availability

The data that support the findings of this study are available in the supplementary material.
