# Supplementary material for: Consensus recommendations for next-generation sequencing-based genetic testing in Rare Neurological diseases
Source: Eur J Hum Genet. 2026 Jul 28;34(10):1503–12. doi: 10.1038/s41431-026-02198-4 (PMC13634049; doi:10.1038/s41431-026-02198-4)
Supplement: Supplementary file 1 [file 41431_2026_2198_MOESM1_ESM.docx]

Supplementary Tables

### **Supplementary Table 1. Members, their stakeholder representation, and assignment to focused working groups**

| **Working Group** | **WG member** | **Stakeholder and professional representation** |
| --- | --- | --- |
| **Methodology (N=7)** | Aleš Maver (coordinator) | Organising team, EuroGentest representative |
|  | Peter Bauer | Sequencing and data-analysis |
|  | Zih-Hua Fang | Sequencing and data-analysis |
|  | Tobias Haack | Sequencing and data-analysis |
|  | Milena Janković | Sequencing and data-analysis |
|  | Marc Sturm | Sequencing and data-analysis |
|  | Rachel Taylor | Quality assurance, EMQN |
| **Interpretation (N=7)** | Katja Lohmann (coordination) | Organising team |
|  | Gabriel Heinz | Clinical variant interpretation |
|  | Erik-Jan Kamsteeg | Clinical variant interpretation |
|  | Kornelia Neveling | Clinical variant interpretation |
|  | Nika Schuermans | Clinical variant interpretation |
|  | Lisenka Vissers | Clinical variant interpretation |
|  | Victoria Williams | Quality assurance, EMQN |
| **Reporting (N=9)** | Holm Graessner (coordination) | Organising team |
|  | Astri Arnesen | Patient representation |
|  | Ivo Barić | Neurology and clinical genetics |
|  | Sylvia Boesch | Professional group representation (EAN neurogenetics) |
|  | Henry Houlden | Professional group representation (EAN neurogenetics) |
|  | Deborah Mascalzoni | Legal representation |
|  | Judit Molnar | Neurology and clinical genetics |
|  | Ivana Novaković | Professional group representation (MDS task group on genetic testing) |
|  | Katie Shiels | Quality assurance, EMQN |
| **Clinical (N=8)** | Whole coordination group | Organising team |
|  | Kailash Bhatia | Professional group representation (EAN coordinating panel on RND) |
|  | Fran Borovečki | Neurology and clinical genetics |
|  | Norbert Brüggemann | Neurology and clinical genetics |
|  | Michelangelo Mancuso | Professional group representation (EAN coordinating panel on RND) |
|  | Alexander Münchau | Neurology and clinical genetics |
|  | Borut Peterlin | Neurology and clinical genetics |
|  | Ludger Schoels | Neurology and clinical genetics |
|  | Marina Tijssen | Neurology and clinical genetics |

# **Supplementary Table 2 - List of topics and outcomes of the first round of the Delphi voting process**

| **ID** | **Topic** | **Average score (range 1-5)** | **Summary score** |
| --- | --- | --- | --- |
| 1 | Definition of a minimal common gene content (panel) for genetic testing of RNDs | 4.3 | 115 |
| 2 | Recommendation on updating the gene panel content | 4.4 | 118 |
| 3 | Handling NGS-related limitations for the genes included in the analysed gene panel | 4.7 | 127 |
| 4 | The recommendation on the use of tests complementary to NGS-based testing | 4.2 | 114 |
| 5 | Recommendations on the minimal set of bioinformatic analyses that should be performed | 4.3 | 117 |
| 6 | The use of extended bioinformatic analyses in the analysis of NGS data | 4.3 | 117 |
| 7 | Provision of data on the bioinformatic algorithms and pipelines used | 3.6 | 98 |
| 8 | Recommendation for strategies and requirements for NGS pipeline validation | 4.4 | 118 |
| 9 | Minimal quality parameters that need to be assessed in the sequencing experiment | 4.4 | 120 |
| 10 | Minimal quality and test parameters that need to be included in the report | 4.0 | 108 |
| 11 | Management of the gaps in sequencing data | 4.2 | 113 |
| 12 | Define broad disease-specific recommendations for NGS-based testing in RNDs | 4.4 | 120 |
| 13 | Address the disease-specific limitations of NGS-based testing | 3.9 | 105 |
| 14 | Disease-specific variant interpretation guidelines | 4.2 | 114 |
| 15 | Recommendations on the use of variant interpretation guidelines | 4.4 | 119 |
| 16 | Systematic reporting of evidence used to classify identified variants | 4.1 | 110 |
| 17 | Sharing variant classifications | 4.2 | 113 |
| 18 | The use of match-making networks to improve the outcomes of patients through data-sharing | 4.0 | 109 |
| 19 | Format and content of the NGS-testing reports | 4.3 | 115 |
| 20 | The use of standard reporting language to minimise ambiguity | 4.5 | 121 |
| 21 | Recommendations on the variants to report | 4.4 | 118 |
| 22 | Define the minimal set of clinical information and guidance to be included in the report | 3.6 | 96 |
| 23 | Recommendations to adjust complexity and information in the report to the target audience (the recipient) | 3.9 | 105 |
| 24 | The clinical context of NGS-based testing | 4.6 | 124 |
| 25 | Reinterpretation of the NGS data | 4 | 109 |
| 26 | Unsolicited findings in NGS-based testing | 4.1 | 111 |
| 27 | Reporting of variants of uncertain significance | 4 | 109 |
| 28 | Recommendations for reporting carrier status | 3.9 | 104 |

# **Supplementary Table 3 - The consensus harmonisation results of the final version of the recommendations**

| Recommendation ID | Recommendation | Average rating (scale 1-10) | Score sum |
| --- | --- | --- | --- |
| R1 | RECOMMENDATION 1/28  NGS-related limitations for the genes included in the analysed gene panel | 9.5 | 123 |
| R2 | RECOMMENDATION 2/28  Minimal set of bioinformatic analyses | 9.4 | 122 |
| R3 | RECOMMENDATION 3/28  The use of extended bioinformatic analyses in the analysis of NGS data | 9.2 | 119 |
| R4 | RECOMMENDATION 4/28  Provision of data on the bioinformatic algorithms and pipelines used | 9.4 | 122 |
| R5 | RECOMMENDATION 5/28  Strategies and requirements for NGS pipeline validation | 9.4 | 122 |
| R6 | RECOMMENDATION 6/28  Minimal quality parameters that need to be assessed in the sequencing experiment | 9.4 | 122 |
| R7 | RECOMMENDATION 7/28  Management of the gaps in sequencing data | 9.4 | 122 |
| R8 | RECOMMENDATION 8/28  Ensuring up-to-date gene panel content | 9.5 | 123 |
| R9 | RECOMMENDATION 9/28  The use of disease-specific variant interpretation guidelines | 9.7 | 126 |
| R10 | RECOMMENDATION 10/28  Consistent use of variant interpretation guidelines | 9.8 | 127 |
| R11 | RECOMMENDATION 11/28  Systematic reporting of evidence used to classify identified variants | 8.7 | 113 |
| R12 | RECOMMENDATION 12/28  Sharing variant classifications | 9.2 | 120 |
| R13 | RECOMMENDATION 13/28  The use of match-making networks to improve the outcomes of patients through data-sharing | 9.4 | 122 |
| R14 | RECOMMENDATION 14/28  Reinterpretation of the NGS data | 9.4 | 122 |
| R15 | RECOMMENDATION 15/28  Format and content of the NGS-testing reports | 8.9 | 116 |
| R16 | RECOMMENDATION 16/28  Minimal quality and test parameters that need to be reported | 9.5 | 124 |
| R17 | RECOMMENDATION 17/28  The use of tests complementary to NGS-based testing | 9.4 | 122 |
| R18 | RECOMMENDATION 18/28  The use of standard reporting language to minimise ambiguity | 9.6 | 125 |
| R19 | RECOMMENDATION 19/28  Selection of variants to include in the NGS-testing report | 8.8 | 115 |
| R20 | RECOMMENDATION 20/28  Unsolicited findings in NGS-based testing | 9.7 | 126 |
| R21 | RECOMMENDATION 21/28  Reporting of variants of uncertain significance | 9.2 | 119 |
| R22 | RECOMMENDATION 22/28  Recommendations to adjust complexity and information in the report to the target audience (the recipient) | 9.2 | 120 |
| R23 | RECOMMENDATION 23/28  Disease-specific recommendations for NGS-based testing in RNDs | 9 | 117 |
| R24 | RECOMMENDATION 24/28  Disease-specific limitations of NGS-based testing | 9.6 | 125 |
| R25 | RECOMMENDATION 25/28  Minimal common gene content (panel) for genetic testing of RNDs | 9.6 | 125 |
| R26 | RECOMMENDATION 26/28  Recommendations for reporting carriership of pathogenic variants | 9.8 | 127 |
| R27 | RECOMMENDATION 27/28  The clinical context of NGS-based testing | 8.2 | 106 |
| R28 | RECOMMENDATION 28/28  Minimal set of clinical information and guidance to be included in the report | 7.3 | 95 |
