# Supplementary material for: Consensus recommendations for next-generation sequencing-based genetic testing in Rare Neurological diseases
Source: Eur J Hum Genet. 2026 Jul 28;34(10):1503–12. doi: 10.1038/s41431-026-02198-4 (PMC13634049; doi:10.1038/s41431-026-02198-4)
Supplement: Supplementary file 2 [file 41431_2026_2198_MOESM2_ESM.docx]

**Supplementary Information**

The best practice recommendations for the use of NGS in diagnosing rare neurologic diseases

Topic 1. **Definition of a minimal common gene content (panel) for genetic testing of RNDs** (Working group Clinical)

**Recommendation**

Laboratories should clearly report the genes assessed and the types of genetic variation covered in their analysis. Given the heterogeneity of rare neurological diseases (RNDs), routinely updated panel resources (e.g. PanelApp) or dynamic gene panel approaches based on phenotype-gene associations (e.g., HPO-based individual panels) should be favored over static, predefined panels. However, laboratories must ensure that at least a core set of high-evidence genes relevant to the referral diagnosis is consistently included. Panels should be regularly updated to reflect current knowledge (see Recommendation on updating the gene panel content), ensuring comprehensive and standardized diagnostic evaluation across institutions. Benchmarking diagnostic performance requires defining minimum gene content, quality standards, and coverage expectations, including CNV detection. While WES/WGS-based approaches offer flexibility, pre-defined, targeted gene panel applications are also possible. Still, target gene panels' utility in RND is limited, and their use is in decline and not advised in the majority of diagnostic situations.

**Clarification of the recommendation/Practical illustration of the issue**

*When analysing the data of NGS-based testing, the laboratories often use in-house panels with variably updated gene content. A clinically relevant finding may, therefore, be missed if the laboratory fails to include a specific gene in their panel despite clear evidence of its causality in the state-of-the-art literature*[^1^](https://www.zotero.org/google-docs/?sioFDF)*. Therefore, the laboratory should ensure that the gene panels are updated and should consider using available solutions to ensure this (i.e. PanelApp). This is necessary to minimize the chance of a patient failing to receive a diagnosis due to a simple issue with gene panels.*

Recommendation 2. **Recommendation on updating the gene panel content** (Working group Interpretation)

**Recommendation**

The gene panel content should be updated at least once a year, but we would recommend doing so every 3-6 months. If virtual panels are used (based on exome or genome sequencing), relevant (frequently mutated) new disease genes should be added immediately as soon as the respective laboratory becomes aware of them. It is important that updates are documented by assigning a new version of the gene panel list and by respective file records. Storing the lists in a secured place is needed to prevent unintended modifications without documentation. Updating the gene lists should be based on a comprehensive literature search, including PubMed, OMIM, PanelApp, and other curated panel information. It is important to carefully evaluate the level of evidence before a new candidate disease gene is included in the panel to avoid false-positive reports. It would be desirable that all diagnostic laboratories use the same curated gene lists per disease. Updating gene panel content should take into account the inclusion of novel disease genes but also the exclusion of genes for which there is accumulated evidence against a gene-disease association.

**Clarification of the recommendation/Practical illustration of the issue**

*A patient with Parkinson´s disease (PD) is referred for gene panel analysis. A negative report is returned by the laboratory based on a targeted gene panel designed in 2023. Notably, this panel does not include screening the RAB32 gene, which was added to the list of PD genes in 2024*[^2,3^](https://www.zotero.org/google-docs/?DhU5Sh)*. The neurologist interprets this report as indicating a non-genetic etiology. However, the patient, in fact, carries a pathogenic variant in RAB32 that was missed due to outdated and incomplete screening. Notably, the variant was inherited to his two offspring who are at increased risk to also develop PD.*

Recommendation 3. **Handling NGS-related limitations for the genes included in the analysed gene panel** (Working group Methodology)

**Recommendation**

It is essential for a laboratory to consistently declare the limitations of the NGS-based testing in the clinical report. It is essential to report when a gene relevant to the phenotype has been analyzed, but a major mutational mechanism for that gene cannot be assessed using the NGS method employed (e.g., repeat expansions in C9orf72 in ALS/FTD). In such cases, the laboratory should either perform an orthogonal test capable of detecting the missing pathogenic variants or clearly state the limitations of the test(s) performed for the gene in an easily visible manner (i.e. first page of the report). Genes that are relevant for the referral diagnosis and have poor coverage should be declared. Also, genes with coverage gaps that may lead to medically significant differences should be declared.

**Clarification of the recommendation/Practical illustration of the issue**

*A patient with chorea is referred for exome sequencing on behalf of the neurologist. A negative report is returned by the laboratory, which does not routinely perform expansion detection in exome data. The neurologist interprets this report as indicating a non-genetic etiology. After a year of detailed diagnostic follow-up and extensive diagnostic evaluation, a re-testing is considered, specifically for Huntington´s disease-associated expansion, returning a positive result. In this example, a clear indication of the possibility of missing the expansion variants and advice for follow-up studies would immediately refer the clinician to do follow-up testing and consider this option, preventing a lengthy and unnecessary diagnostic work-up.*

Recommendation 4. **The recommendation on the use of tests complementary to NGS-based testing** (Working group Reporting)

**Recommendation**

NGS test limitations, such as the inability to detect repeat expansions or large CNVs, should be clearly stated in reports. Clinicians should be guided on appropriate follow-up testing, with explicit recommendations when additional analyses, such as FMR1 repeat expansion testing, are relevant. If a required complementary test is unavailable, clinicians should be informed of the test's limitations and possible referral options. Testing recommendations should be phenotype-driven, ensuring appropriate methods are suggested based on disease mechanisms (e.g., repeat-primed PCR for CANVAS syndrome). Alternative technologies, such as optical genome mapping, should be considered when NGS is insufficient. Finally, all recommendations should be integrated into reports and discussed during genetic counseling to support clinical decision-making.

**Clarification of the recommendation/Practical illustration of the issue**

*A 59-year old patient with suspected CANVAS is referred to expanded genetic testing using exome sequencing by the referring neurologist. The laboratory returns a negative exome sequencing result without an indication that exome sequencing is not an appropriate method to reliably ascertain presence of the RFC1 expansion - the laboratory includes a generic statement in details of the report that expansions can not be reliably ascertained by the method. The clinician receives a negative report without clear guidance for follow-up testing, leading to the delay in molecular diagnosis of the disease. This case illustrates the necessity to clearly indicate the guidance for the recipient of the report to proceed with follow-up testing if the NGS-based test is an inappropriate test to diagnose the primary referral. This limitation should be clearly stated in the primary conclusions of the report - a disclaimer in the details may not be sufficient to properly inform the recipient of the required follow-up testing to ensure appropriate diagnostic procedure.*

Recommendation 5. **Recommendations on the minimal set of bioinformatic analyses that should be performed** (Working group Methodology)

**Recommendation**

There is a wide variability of bioinformatic approaches used by the laboratories. While the implementation differs across laboratories, a minimum set of bioinformatic algorithms should be performed in every NGS-based diagnostic test. This minimum set of bioinformatic approaches should include

* approaches for quality control of raw sequencing data,

* alignment to a recent version of the human genome,

* quality assessment of the coverage across declared clinical targets,

* variant calling with a variant caller with demonstrated high sensitivity and specificity, and

* assessment of the quality of the variant calling process.

**Clarification of the recommendation/Practical illustration of the issue**

*The laboratory performs whole-genome sequencing (WGS) in a patient with spastic paraplegia. They only identify a single heterozygous pathogenic SPG7 variant, which is insufficient to causally clarify the disease. The laboratory uses generic wording for the quality assessment of the report, stating that >98% of regions are covered. After recontacting the laboratory, it is made evident that the coverage of the WGS experiment was relatively low (26x), leading to a relatively poorly covered region in the 1st exon of the SPG7 gene, which was missed by WGS due to low overall coverage. A clear provision of the actual experiment's quality parameters is thus essential to guide follow-up genetic testing and decision-making.*

Recommendation 6. **The use of extended bioinformatic analyses in the analysis of NGS data** (Working group Methodology)

**Recommendation**

In addition to SNV detection, NGS-based testing enables a wide assortment of extended data analyses, which allow for the interrogation of other mutational mechanisms, including copy number variants, structural variants, and expansions.

Given the variable utilization and variability of bioinformatic approaches to perform extended NGS data analyses, a testing report should clearly indicate which types of variants in addition to SNV were investigated and which algorithms were used.

Given the significance of pathogenic copy number variants (CNV) in human monogenic disorders and the general availability of multiple efficient algorithms for CNV detection, this analysis should be performed as part of the routine analysis of NGS-based approaches sequencing data. This is of particular importance in conditions where copy number variants prevail as a mechanism of pathogenicity (i.e. exon deletions and duplication in the PRKN gene associated with Parkinson's disease).

When using approaches to detect non-SNV variation, the laboratory should perform internal validation to ensure appropriate sensitivity or utilize a pre-validated variant detection algorithm. It is strongly advisable to report these parameters in the details of the report.

When using extended bioinformatic approaches, the laboratory should advise confirmatory methods, unless the validity of the method has been clearly demonstrated.

Some approaches are highly related to the sequencing quality and inter-sample variability in the performance of the extended bioinformatic algorithms. It is advisable to offer information on the performance of the algorithm in the currently performed experiment.

When using extended bioinformatic approaches, the laboratory should advise confirmatory methods, unless the validity of the method has been clearly demonstrated.

**Clarification of the recommendation/Practical illustration of the issue**

*A patient with an early onset of Parkinson's disease is referred for genetic testing using exome sequencing. The laboratory reported a single heterozygous pathogenic loss-of-function variant in the PRKN gene, clearly stating that this finding is insufficient to establish a genetic diagnosis, due to recessive inheritance of PRKN-associated Parkinson's disease. The laboratory does report using a CNV-calling algorithm, but they have not validated it for single exon deletions/duplications. The patient remains undiagnosed. In this case, it would be advisable for the laboratory to clearly indicate and refer the patient to follow-up testing and to provide information on the sensitivity for detection of copy number variants, particularly given the large prevalence of exon deletions and duplications observed in the PRKN gene. It is not sufficient to only have a general disclaimer in the details of the report. Such limitations should be contextual to the referral and findings and should be highlighted in the principal findings and/or limitations of the report.*

Recommendation 7. **Provision of data on the bioinformatic algorithms and pipelines used** (Working group Methodology)

**Recommendation**

Data generated by NGS-based diagnostic testing is analysed using a wide variety of computational algorithms implemented within diverse software tools. This may lead to clinically significant differences in variant detection. For this reason, at minimum, basic information on the bioinformatic tools and approaches used to analyse data should be included in the report.

The NGS-based diagnostic reports should include information on the tools for read alignment, variant calling and variant annotation. An additional essential piece of information that should be provided is the reference genome version and resources or databases used by the algorithms.

Reference to accessible, reproducible and versioned code repositories with the pipelines used in the procedure is strongly encouraged (ie. Github).

**Clarification of the recommendation/Practical illustration of the issue**

*A laboratory is performing a diagnosis in a patient with suspected MELAS syndrome. Their pipeline uses a hard cutoff for filtering out the variants with an allelic ratio below 10%, and they release a negative report. After years of work-up, the patient has several strokes, further raising suspicion for MELAS. A sample of another tissue is sent for sequencing, revealing the presence of the recurrent pathogenic MELAS variant. A revision of the original raw data indicates that the variant was visible at that point already but has been filtered out by the pipeline. In this case, the clear provision of the analytical steps could allow one to consider the analytical steps to check whether low-heteroplasmic variants, frequent in MELAS, were considered in the analysis.*

Topic 8. **Recommendation for strategies and requirements for NGS pipeline validation** (Working group Methodology)

**Recommendation**

The analytical pipeline should be routinely validated and sensitivity/specificity for SNV detection and and for other mutational types. There are multiple available strategies to achieve this with internal and external quality controls. At minimum, the laboratory should perform in-house pipeline validation using a reference sample or a reference dataset (ie. genome in a bottle). An alternative approach is to participate in the external quality assessment schemes that ascertain the sensitivity and specificity of the data pipelines.

**Clarification of the recommendation/Practical illustration of the issue**

*A patient with suspected dystonia has been tested using exome sequencing. The laboratory has been using a pipeline, where a variant analysis step resulted in poor detection of indels, leading to a missed diagnosis. A routine pipeline validation process could reveal this issue and prevent the missed diagnosis.*

Recommendation 9. **Minimal quality parameters that need to be assessed in the sequencing experiment** (Working group Methodology)

**Recommendation**

There is a wide variability in depth and breadth of sequencing approaches used by the laboratories offering NGS-based testing. Therefore it is essential that laboratories clearly report the parameters of quality of the sequencing experiment. This allows for critical assessment of diagnostic results and may guide follow-up testing. At minimum, the laboratories should provide the calculated metrics of the depth of coverage and the proportion of sequencing targets covered with a minimum defined coverage. This allows for general assessment of the quality of the sequencing experiment and allows one to assess the likelihood of coverage gaps impacting the variant detection. The target tested should be clearly defined (ie. a set of coding regions of a list of genes that are sequenced should be provided and it should be distinguished from the target considered in the interpretation).

**Clarification of the recommendation/Practical illustration of the issue**

*There is a large variability in coverage thresholds that laboratories deem clinically valid, which mandates the consistent reporting of these QC metrics. For example, a laboratory performing genome sequencing at 30x may miss variants that would be detectable by genome sequencing at 60x coverage. Further, the median coverage itself does not ensure consistent coverage across the genome. For example, in a given exome with median coverage of 100x, some critical regions may still not be covered sufficiently if the capture is not successful. This would be reflected by the percentage of regions covered with the callable 20x coverage being lower than acceptable. While the move towards whole genome sequencing and improvements to exome capture protocols have greatly reduced the sequencing gaps in NGS-based tests, there are still regions which are inherently difficult to analyse using short-read sequencing approaches.*

Recommendation 10. **Minimal quality and test parameters that need to be included in the report** (Working group Reporting)

**Recommendation**

NGS reports should clearly state essential details, including the sequencing platform, capture method, read length, and other parameters affecting data quality. Depth of coverage and percentage of target regions covered at a predefined threshold must be reported, with minimal QC standards established and documented. While full QC metrics are necessary for accreditation and internal validation, only key quality parameters should be included in reports, with detailed data available upon request. Reports should maintain uniformity across laboratories to ensure consistency. Clinician-focused reporting should balance transparency with readability, avoiding unnecessary complexity while retaining essential information for potential future reanalysis.

**Clarification of the recommendation/Practical illustration of the issue**

*A patient with dystonia is referred for diagnostic re-evaluation after a negative exome sequencing report performed in 2013. The laboratory requests the data based on which the original report was prepared. A reanalysis of data reveals a non-homogenous coverage with over 5% of regions covered below 10x. Repeat exome sequencing reveals presence of a pathogenic variant in the first exon of the GCH1 gene, leading to a diagnosis of dystonia with possibility of imminent interventions. Although the technical details of genetic testing may be overwhelming for the clinician and the patient, the minimal quality criteria of sequencing should be clearly stated, to account for differences across the methodologies used at different institutions. This is also necessary for potential re-assessment of sequencing data in patients without diagnosis, facilitating potential decisions for re-testing or further genetic testing.*

Recommendation 11. **Management of the gaps in sequencing data** (Working group Methodology)

**Recommendation**

The laboratory should clearly indicate if there is a possibility of the existence of gaps in the sequencing data that could impact a diagnostic result. The laboratory should have an established strategy for handling gaps in sequencing data and should declare its strategy for managing gaps in the clinical report. If the referral has a high a priori probability for a disorder associated with a specific gene and a gap is present in that gene, the laboratory should either sequence the region missing with a separate test or they should clearly indicate that the region has not been covered in the report, mentioning that follow-up testing is necessary.

**Clarification of the recommendation/Practical illustration of the issue**

*A child with a suspected hypomyelinating leukodystrophy is referred for genetic testing and the laboratory issues a negative report. A promoter variant in the UFM1 gene is a frequent cause of leukodystropy in the geographic region, from which the patient originates. The laboratory did list the UFM1 gene in the panel, giving the impression that the most relevant finding in this gene has been assessed and not being present. When there is a high likelihood of a gap in sequencing leading to false negative results, the laboratory should indicate this in the limitations of the test and indicate follow-up testing.*

Recommendation 12. **Define broad disease-specific recommendations for NGS-based testing in RNDs** (Working group Clinical)

**Recommendation**

NGS should be positioned appropriately within the diagnostic workflow for rare neurological diseases (RNDs), with clear guidelines on when to use exome (WES), genome (WGS), or targeted approaches. Alternative methods should be recommended when NGS does not adequately detect specific variants (e.g., triplet repeat expansions, structural variants, mtDNA heteroplasmy). Reports should include diagnostic yield estimates and highlight genes where standard NGS has limitations. A common framework for prioritizing frequent disease-associated variants (e.g., C9orf72, FGF14) would improve standardization, ensuring clinicians make informed decisions about genetic testing.

**Clarification of the recommendation/Practical illustration of the issue**

*While a patient with unspecified ataxia may benefit from early whole genome sequencing with the ability to capture small sequence and copy number variants, complex structural variation, including repeat expansions in RFC1, will be missed by a short-read NGS-based approach. This would be specifically relevant in a patient with the diagnostic suspicion of CANVAS syndrome due to the limited diagnostic capacity of NGS to diagnose expansion disorders.*

Recommendation 13. **Address the disease-specific limitations of NGS-based testing** (Working group Reporting & Clinical)

**Recommendation**

A remarkable diversity of genetic mutational mechanisms characterizes rare neurological diseases (RND). While NGS-based testing is developing towards becoming a single comprehensive test in RND patients, laboratories should handle its limitations appropriately. Failure to clearly report the inability to detect certain mutational mechanisms (e.g., structural variants and expansions) may result in a delay or even miss the diagnosis and, consequently, lead to patient harm. The laboratory should perform genetic testing and reporting in the context of the current patient. If the patient is referred for NGS-based testing for a condition associated with a genetic etiology that can not be reliably detected or is not ascertained by the current laboratory, this should be clearly stated in the conclusions of the report, and guidance should be given on what test should be used to complement NGS-based testing.

**Clarification of the recommendation/Practical illustration of the issue**

*The current literature indicates that NGS-based testing has a high sensitivity for detecting expansion-related disorders (97.3%*[^5^](https://www.zotero.org/google-docs/?eeXCkM)*). However, this is not sufficient for situations, where there is a high a priori probability for a specific expansion disorder (ie. Huntington disease). Furthermore, expansion detection based on short-read sequencing data is still limited in accurately determining the size of repeats, which is crucial in determining the disease prognosis, outcomes and reproductive decisions.*

Recommendation 14. **Disease-specific variant interpretation guidelines** (Working group Interpretation)

**Recommendation**

Disease-specific variant interpretation guidelines are relevant for repeat expansion disorders that cause a variety of rare neurological disorders. For the interpretation of repeat expansions, the disease-causing number of repeats depends on the gene. Interruptions of the repeated sequence motif often, but not always, decrease pathogenicity. Respective information needs to be retrieved from comprehensive and recent review articles or by reviewing the available literature. There are usually different levels of penetrance depending on the repeat size and structure, including age-dependent effects. Further, it is important to indicate whether repeat expansions have been targeted with the used assay in negative testing reports. Many repeat expansions are not covered by short-read next-generation sequencing approaches.

**Clarification of the recommendation/Practical illustration of the issue**

*In addition to missed repeat expansions, for instance, in the HTT or FGF14 gene (see below), the relevance of correct repeat-length interpretation is illustrated here by a real-world example from the literature*[^6^](https://www.zotero.org/google-docs/?afw5kG)*. A seemingly healthy mother with a repeat expansion in the FMR1 gene in the premutation range (77 CGG) had a son with a longer repeat (103 CGG) and a daughter with a massively expanded, unstable allele (220-1822 CGG). The son was healthy until the age of 57 years, when he developed tremor, followed 8 years later by gait ataxia, word-finding difficulties, and slurred speech. A diagnosis of fragile X-associated tremor/ataxia syndrome (FXTAS) was made. In contrast, the daughter had intellectual disability compatible with a diagnosis of fragile X syndrome (FXS). Thus, different repeat lengths in this family are associated with different phenotypic findings that genetic testing laboratories need to be aware of.*

Recommendation 15. **Recommendations on the use of variant interpretation guidelines** (Working group Interpretation)

**Recommendation**

The universal variant classification (ACMG guideline) should be used for all variant interpretations. Usage of an in-house system is also acceptable, but the ratings need to be explained in detail and it should follow the main principles of the ACMG criteria. Variant interpretation should not only rely on ClinVar, especially if the confidence rating is low (one- or two-star entries which indicate entries that have not been reviewed by a variant curation expert panel [VCEP]). Caution should be used when applying automatic scoring systems based on the ACMG criteria such as Varsome and Franklin. There is often an overinterpretation of truncating variants since the PVS1 criterion is applied for almost all genes, even if truncating variants are not clearly a disease cause and the mechanism is rather a gain of function. Also in these cases, expert knowledge is needed, and clinical interpretation should be an essential part of the variant interpretation process. Building such expert panels needs to be fostered and can be based on the ClinGen infrastructure supporting VCEPs. The outcome should also be distributed by systematic reviews and incorporated in databases such as the widely used and accepted ClinVar database.

**Clarification of the recommendation/Practical illustration of the issue**

*Heterozygous pathogenic variants in LRRK2 are a relatively frequent cause of Parkinson´s disease (PD). The disease mechanism seems to be an increase in LRRK2´s kinase activity caused by a variety of missense variants, while heterozygous truncating variants have not been linked to PD*[^7^](https://www.zotero.org/google-docs/?TIURJy)*. However, pathogenicity scoring using Varsome interprets a previously reported truncating variant (c.6187_6191delCTCTA) as likely pathogenic by wrongly applying the PVS1 criterion (reviewed in* [^8^](https://www.zotero.org/google-docs/?gFcEEA)*).*

Recommendation 16. **Systematic reporting of evidence used to classify identified variants** (Working group Interpretation)

**Recommendation**

The used ACMG criteria (e.g. PS1, PM1 etc.) for the variant classification should be mentioned in the report, but plain language is recommended over abbreviations or only providing the ACMG codes (e.g. PS1, PM1 etc.). Instead, meaningful information such as the frequency in controls and patients, in-silico prediction outcomes, conservation across species in case of missense variants, positive functional read-outs, and information on segregation and zygosity should be provided. Pitfalls need to be considered, including population-specific frequencies, especially in inbred populations, limitations of in-silico predictions, and the variety of functional tests (a negative functional test might not rule out that a different than the tested protein function is disturbed). It is also important to indicate if the variant has previously been published as disease-causing. Reference to respective literature, for instance, by using PMIDs should be provided. If there are only few reports, all links should be listed, but for well-established pathogenic variants reference to the original publication and a recent review article is considered sufficient.

**Clarification of the recommendation/Practical illustration of the issue**

*The location of a variant in a protein may be specifically relevant when different isoforms of the protein exist and functional testing needs to take into account which isoform is affected. As an example, hereditary spastic paraplegia (HSP) is often caused by heterozygous, pathogenic variants in the SPAST gene. Interestingly, SPAST encodes two major spastin isoforms, M1- and M87-spastin, which are generated from distinct translation initiation sites. There is evidence of a dual disease mechanism involving both haploinsufficiency, characterized by reduced functional spastin levels, and a gain-of-toxicity mechanism, driven by the accumulation of mutant spastin. Notably, the few yet detected variants that only affect M1 seem to be linked to a milder phenotype*[^9^](https://www.zotero.org/google-docs/?9oOC8O)*.*

Recommendation 17. **Sharing variant classifications** (Working group Interpretation)

**Recommendation**

We recommend sharing variant interpretations within the diagnostic and research community. This is especially relevant for variants of uncertain significance (VUS) since cumulative evidence of pathogenicity can provide further insights and lead to an upgrade of the interpretation. Although resources are limited, we recommend undertaking efforts to include sharing of variant classifications, for instance, via ClinVar, in the workflow. If possible phenotypic information should also be provided.

**Clarification of the recommendation/Practical illustration of the issue**

*In the literature, numerous examples exist where a VUS was upgraded to a pathogenic variant through collaborative efforts. For instance, we have been following a patient with early-onset, levodopa-responsive tremor–parkinsonism syndrome for many years. The initial genetic testing in 2018 revealed compound heterozygous variants in the WARS2 gene, including a missense variant that was only considered a VUS. Since then, several additional patients have been reported, expanding the initially linked phenotype of a predominant neurodevelopmental phenotype with developmental delay and intellectual disability, with only mild movement disorders, to prominent movement disorders such as tremor and parkinsonism with only mild or no intellectual disability. The identification of additional carriers of the same missense variant and overlapping phenotypes (as well as functional tests) then revealed a pathogenic role of this variant in our and the other patients*[^10^](https://www.zotero.org/google-docs/?u9zXi3)*. Thus, exchanging VUS and collecting cumulative evidence (or not) can guide variant interpretation.*

Recommendation 18. **The use of match-making networks to improve the outcomes of patients through data-sharing** (Working group Interpretation)

**Recommendation**

In addition to posting variant interpretations on ClinVar, match-making could become an important part of the variant interpretation workflow. This is another way of sharing and improving variant classifications together to benefit the patients and their families. It would be of great assistance for variants with uncertain interpretation, i.e., VUS. This approach is essential for variants in genes that are molecularly targetable/treatable disorders. Current limitations for a widespread application of match-making include limited resources and potentially ethical issues such as the requirement of patient consent.

**Clarification of the recommendation/Practical illustration of the issue**

*Match-making tools such as GeneMatcher are widely used in the research community, especially in the field of neurodevelopmental disorders where it helped to validate the disease-causing role of variants in many genes. In the field of RND, match-making was used to establish VPS13D as a novel gene for spastic ataxia*[^11^](https://www.zotero.org/google-docs/?dIWBJ2)*.*

Recommendation 19. **Format and content of the NGS-testing reports** (Working group Reporting)

**Recommendation**

NGS reports should follow a clear, structured, and standardized format to ensure ease of interpretation while remaining concise yet comprehensive. A uniform reporting structure, at least at a national level, should be encouraged, aligning with established guidelines like ESHG and ISO 15189. Reports must be designed to prevent misinterpretation, with key findings clearly highlighted. Standardized formatting enhances clinical communication, making reports more comparable across laboratories. While consistency is essential, some flexibility should be allowed to accommodate different reporting styles and workflows, ensuring reports remain both standardized and practical for clinical use.

**Clarification of the recommendation/Practical illustration of the issue**

*A clinician receives a report of next-generation sequencing that is 7 pages long with the principal finding reported on 3rd page of the report, followed by multiple variants of uncertain significance reported on page 4. Based on the clinical overlap the clinician sees one of the variants of uncertain significance as the most fitting finding, leading to an entry of an incorrect diagnosis in the medical health record of the patient, which results in inappropriate management of the patient at several other specialists. Such cases illustrate the need for concise reporting despite the underlying complexity of the NGS-based tests, having the recipient of the report in mind. Therefore, the principal finding, relevant to the referral condition should be clearly outlined in the initial sections of the report and the care should be taken to minimise the impact of other information on the clarity of report.*

Recommendation 20. **The use of standard reporting language to minimise ambiguity** (Working group Reporting)

**Recommendation**

NGS reports should use standardized and well-defined terminology to ensure clarity and consistency while allowing flexibility for different levels of evidence. Findings must be clearly linked to the patient’s referral indication, with transparent reporting of uncertain results and guidance on follow-up actions. Consistency across laboratories and languages is essential, with adherence to internationally recognized guidelines (e.g., ACMG, HGVS) to improve communication and support cross-border collaborations. While harmonized language is important, laboratories should retain flexibility to adapt statements to specific clinical contexts, ensuring reports remain informative and actionable.

**Clarification of the recommendation/Practical illustration of the issue**

*A 71-year old patient with frontotemporal dementia is referred for whole genome sequencing. A variant of uncertain significance in MAPT gene is reported as a potential cause of the clinical presentation in the patient, without clearly stating the uncertainty of the finding until further evidence is available. Upon receiving the reports the relatives request pre-symptomatic testing for this variant, stating that the possible cause has been identified in their parent. To minimise the misunderstanding between laboratories and recipients of the report, it is strongly advised that the laboratory attempts to use a standardised reporting style, aligning with best practice guidelines (an example is the ACGS guidelines). A standardised reporting style minimises the chance of confusion due to different understanding of the content and ensures harmonisation across different institutions.*

Recommendation 21. **Recommendations on the variants to report** (Working group Reporting)

**Recommendation**

NGS reports should clearly define which variants are included, prioritizing clinically relevant findings while avoiding non-actionable or confusing results. VUS reporting should be context-specific, with ‘hot’ VUS included only when emerging evidence suggests pathogenicity, while unnecessary reporting should be avoided. Standardization is key, but flexibility should allow for disease-specific exceptions, following international guidelines (ACMG, ESHG, ClinGen). Clinically irrelevant findings, including high-frequency recessive carrier status, should be excluded to prevent misinterpretation. Clinician and patient preferences may influence reporting, emphasizing the need for transparent communication between laboratories and healthcare providers.

**Clarification of the recommendation/Practical illustration of the issue**

*There are existing recommendations for reporting of variants in genome sequencing that limit the reporting to (likely) pathogenic variants and variants of uncertain significance that are clinically consistent with referral and for which additional steps can be performed to clarify their impact*[^12^](https://www.zotero.org/google-docs/?BSK0zh)*, which aligns with the present recommendation.*

Recommendation 22. **Define the minimal set of clinical information and guidance to be included in the report** (Working group Clinical)

**Recommendation**

NGS reports should be standalone documents that provide essential clinical guidance while respecting the roles of laboratories and treating physicians. Genetic counseling should be consistently recommended, particularly when a genetic diagnosis is made. If additional testing (e.g., segregation analysis) is needed to confirm diagnostic relevance, this should be clearly advised. While treatment decisions remain the clinician's responsibility, reports should indicate the possibility of treatments if they are available in the health care system, refer the patient to expert networks (e.g., ERNs), and advise management according to state-of-the-art clinical management guidance. Standardization across laboratories will improve clarity, communication, and patient care.

**Clarification of the recommendation/Practical illustration of the issue**

*When a patient and the healthcare professional receive the genetic testing report, it may be challenging to determine the relevance of the finding for downstream management. For example, if a patient with dystonia receives a report with a variant in the GCH1 gene, the laboratory should clarify that follow-up sequencing in parental samples is necessary to support clarification of the variant if its significance is uncertain, that the penetrance of GCH1-associated dystonia is dependent on the gender and that a possible treatment is available for the disease*[^13^](https://www.zotero.org/google-docs/?fAfKmZ)*.*

Recommendation 23. **Recommendations to adjust complexity and information in the report to the target audience (the recipient)** (Working group Reporting)

**Recommendation**

NGS reports should provide a concise summary of key findings on the first page, ensuring clinicians can quickly grasp results and next steps. Reports should be structured for both geneticists and non-genetic specialists, with brief explanations or references for complex terminology. Balancing readability with scientific accuracy is essential to maintain clarity without oversimplifying critical details. While clinical geneticists play a key role in post-test counseling, reports should be standalone documents that are usable across institutions. Standardized formatting should be prioritized, with detailed technical data stored separately for access when needed. Reports should remain clinician-focused, avoiding oversimplification that could compromise medical accuracy.

**Clarification of the recommendation/Practical illustration of the issue**

*The laboratories should consider including essential guidance and information on the mode of inheritance, recurrence risk and imminent therapeutic interventions that could impact the management of the patient.*

Recommendation 24. **Reinterpretation of the NGS data** (Working group Interpretation)

**Recommendation**

Reinterpretation of NGS data can lead to new diagnoses by taking new evidence into account. There are two layers of re-interpretation, which can be on the gene level (variants in newly identified disease genes) and on the variant level (updates of variant interpretation). The latter one is of great importance for VUS. A fixed schedule for re-interpretation of all VUSs and gene panels every two years would be desirable, but given the workload, it might not be feasible everywhere. A bioinformatic reinterpretation pipeline based on AI technology may be a future tool to assist with re-interpretation to regularly prove changes in the variant classification automatically. A new updated report has to be issued in cases of a reclassification of variants when it leads to a different clinical interpretation. It is crucial that in the current (first) reports, it is clearly indicated that variant interpretation is based on current knowledge and represents only a "snapshot", and that later reinterpretation of variants may provide further insights. Challenges for variant interpretation also include questions about patients´ consent.

**Clarification of the recommendation/Practical illustration of the issue**

*The power of a (systematic) re-analysis was recently demonstrated by the Solve-RD consortium where a Pan-European reanalysis eventually revealed a molecular diagnosis in 12.6% of the more than 6000 reanalyzed patients*[^18^](https://www.zotero.org/google-docs/?aPK3Ay)*, underlining the power of reanalyses.*

Recommendation 25. **Unsolicited findings in NGS-based testing** (Working group Reporting)

**Recommendation**

Patient consent is essential—secondary findings should only be reported if the patient has explicitly opted in during pre-test counseling, with preferences documented for future reanalysis. Only clinically relevant and actionable findings should be reported, following established guidelines (e.g., ACMG), while VUS should generally be excluded to avoid unnecessary anxiety. To minimize harm, virtual panels can help limit unwanted findings, and clear protocols should ensure proper genetic counseling. Compliance with national and international regulations is necessary, and standardized protocols across laboratories would improve consistency in managing and reporting unsolicited findings.

**Clarification of the recommendation/Practical illustration of the issue**

*The patient and the professionals should be appropriately informed that the novel genetic testing approaches also bring about the possibility of identifying secondary findings unrelated to the referral diagnosis. For this reason, NGS-based approaches should be handled specifically to ensure the appropriate informing and consenting process, with the health care system ensuring follow-up counseling and management for patients*[^19^](https://www.zotero.org/google-docs/?YZjsgK)*.*

Recommendation 26. **Reporting of variants of uncertain significance** (Working group Reporting)

**Recommendation**

Laboratories should establish clear criteria for VUS reporting, limiting it to cases where variants are relevant to the clinical indication or have strong disease associations. Country-specific regulations must be followed, with policies on VUS reporting clearly stated in reports. Reporting should be context-dependent, considering both potential benefits (future reclassification) and risks (misinterpretation, anxiety). Unnecessary VUS reporting should be avoided to prevent diagnostic confusion, and genetic counseling is recommended when a VUS is included. Long-term monitoring and reclassification of VUS should be in place, with clinicians and patients informed of possible updates. An alternative approach could involve reporting VUS separately, only if the patient explicitly consents.

**Clarification of the recommendation/Practical illustration of the issue**

*A patient with hereditary spastic paraplegia was referred for exome sequencing and received a negative report. After several years a reinterpretation was ordered, revealing a likely pathogenic variant in SPAST gene, affecting intronic splicing. Retrospective re-assessment variant has shown could have been reclassified based on segregation studies in parental samples after the first analysis, however, it was the policy of the laboratory not to report VUS findings. Although the studies indicate highly variable practices in reporting VUS findings*[^20^](https://www.zotero.org/google-docs/?YK8yrC) *it is important for the laboratory to clearly define what their policies on reporting VUS findings are in the report, making it clear that potential clinically relevant findings are not reported. When a relatively straightforward follow-up testing or clinical assessment could result in reclassification, we believe that this should be indicated in the report to alert the recipient of the possible steps to reach the diagnosis.*

Recommendation 27. **Recommendations for reporting carrier status** (Working group Clinical)

**Recommendation**

Carrier status should be reported, but only when relevant to the clinical indication. Heterozygous variants in recessively inherited conditions should be included, especially when an expected second variant was not found in a gene consistent with the patient’s phenotype. For secondary findings, reporting should be limited to genes recommended for disclosure and should follow patient consent preferences. Carrier status should generally not be reported for unrelated conditions, except in reproductive counseling, where findings in both partners may be relevant. Ethical considerations and informed consent should guide reporting, ensuring patients receive appropriate information without unnecessary stress. Additionally, genetic counselling is advised to ensure appropriate guidance to the patient regarding these findings.

**Clarification of the recommendation/Practical illustration of the issue**

*A 4-year-old patient who had normal early development and walked independently at the age of 14 months was referred for genetic testing since her parents noted clumsiness and frequent falls since she was 3 years old. A neurological assessment detected possible early signs of ataxia, such as an unsteady gait and mild dysarthria. Comprehensive exome sequencing, including CNV analysis, as well as repeat expansion analysis in known SCA genes, did not reveal a genetic cause. Only a heterozygous, but known pathogenic missense variant in the NPC1 gene was detected and reported. The referring child pediatrician thought that Niemann-Pick disease would be an excellent match and initiated NPC1 Sanger sequencing, which revealed a by NGS hard-to-detect splice region variant on the other allele in the patient. This variant was missed by NGS because it was located in a homopolymer stretch*[^21^](https://www.zotero.org/google-docs/?wCmogk)*. Migustat therapy was offered to the patient*[^22^](https://www.zotero.org/google-docs/?A3Prck)*.*

# References

[1](https://www.zotero.org/google-docs/?MlHSAz)	[Maver A *et al.* Quality assurance for next-generation sequencing diagnostics of rare neurological diseases in the European Reference Network. *Eur J Hum Genet* 2024; **32**: 1014–1021.](https://www.zotero.org/google-docs/?MlHSAz)

[2](https://www.zotero.org/google-docs/?MlHSAz)	[Gustavsson EK *et al.* RAB32 Ser71Arg in autosomal dominant Parkinson’s disease: linkage, association, and functional analyses. *Lancet Neurol* 2024; **23**: 603–614.](https://www.zotero.org/google-docs/?MlHSAz)

[3](https://www.zotero.org/google-docs/?MlHSAz)	[Hop PJ *et al.* Systematic rare variant analyses identify RAB32 as a susceptibility gene for familial Parkinson’s disease. *Nat Genet* 2024; **56**: 1371–1376.](https://www.zotero.org/google-docs/?MlHSAz)

[4](https://www.zotero.org/google-docs/?MlHSAz)	[Ryan NM, Corvin A. Investigating the dark-side of the genome: a barrier to human disease variant discovery? *Biol Res* 2023; **56**: 42.](https://www.zotero.org/google-docs/?MlHSAz)

[5](https://www.zotero.org/google-docs/?MlHSAz)	[Ibañez K *et al.* Whole genome sequencing for the diagnosis of neurological repeat expansion disorders in the UK: a retrospective diagnostic accuracy and prospective clinical validation study. *Lancet Neurol* 2022; **21**: 234–245.](https://www.zotero.org/google-docs/?MlHSAz)

[6](https://www.zotero.org/google-docs/?MlHSAz)	[Bartlett E *et al.* Paternal retraction of a fragile X allele to normal size, showing normal function over two generations. *Am J Med Genet A* 2022; **188**: 304–309.](https://www.zotero.org/google-docs/?MlHSAz)

[7](https://www.zotero.org/google-docs/?MlHSAz)	[Beetz C *et al.* LRRK2 Loss-of-Function Variants in Patients with Rare Diseases: No Evidence for a Phenotypic Impact. *Mov Disord Off J Mov Disord Soc* 2021; **36**: 1029–1031.](https://www.zotero.org/google-docs/?MlHSAz)

[8](https://www.zotero.org/google-docs/?MlHSAz)	[Krüger C *et al.* Updated MDSGene review on the clinical and genetic spectrum of LRRK2 variants in Parkinson´s disease. *NPJ Park Dis* 2025; **11**: 30.](https://www.zotero.org/google-docs/?MlHSAz)

[9](https://www.zotero.org/google-docs/?MlHSAz)	[Ramakrishnan S, Mohan N, Dong Z, Liu M, Qiang L. Unraveling Isoform Complexity: The Roles of M1- and M87-Spastin in Spastic Paraplegia 4 (SPG4). *Mov Disord Off J Mov Disord Soc* 2025; **40**: 420–430.](https://www.zotero.org/google-docs/?MlHSAz)

[10](https://www.zotero.org/google-docs/?MlHSAz)	[Pauly MG *et al.* The Expanding Phenotypical Spectrum of WARS2-Related Disorder: Four Novel Cases with a Common Recurrent Variant. *Genes* 2023; **14**: 822.](https://www.zotero.org/google-docs/?MlHSAz)

[11](https://www.zotero.org/google-docs/?MlHSAz)	[Seong E *et al.* Mutations in VPS13D lead to a new recessive ataxia with spasticity and mitochondrial defects. *Ann Neurol* 2018; **83**: 1075–1088.](https://www.zotero.org/google-docs/?MlHSAz)

[12](https://www.zotero.org/google-docs/?MlHSAz)	[Souche E *et al.* Recommendations for whole genome sequencing in diagnostics for rare diseases. *Eur J Hum Genet* 2022; **30**: 1017–1021.](https://www.zotero.org/google-docs/?MlHSAz)

[13](https://www.zotero.org/google-docs/?MlHSAz)	[Weissbach A *et al.* Relationship of Genotype, Phenotype, and Treatment in Dopa-Responsive Dystonia: MDSGene Review. *Mov Disord Off J Mov Disord Soc* 2022; **37**: 237–252.](https://www.zotero.org/google-docs/?MlHSAz)

[14](https://www.zotero.org/google-docs/?MlHSAz)	[Thomsen M *et al.* Genetic Diversity and Expanded Phenotypes in Dystonia: Insights From Large-Scale Exome Sequencing. *Ann Clin Transl Neurol* 2025; **12**: 1648–1659.](https://www.zotero.org/google-docs/?MlHSAz)

[15](https://www.zotero.org/google-docs/?MlHSAz)	[den Hollander B *et al.* The Metabolic Treatabolome and Inborn Errors of Metabolism Knowledgebase therapy tool: Do not miss the opportunity to treat! *J Inherit Metab Dis* 2025; **48**: e12835.](https://www.zotero.org/google-docs/?MlHSAz)

[16](https://www.zotero.org/google-docs/?MlHSAz)	[Hoytema van Konijnenburg EMM *et al.* Treatable inherited metabolic disorders causing intellectual disability: 2021 review and digital app. *Orphanet J Rare Dis* 2021; **16**: 170.](https://www.zotero.org/google-docs/?MlHSAz)

[17](https://www.zotero.org/google-docs/?MlHSAz)	[Jinnah HA *et al.* Treatable inherited rare movement disorders. *Mov Disord Off J Mov Disord Soc* 2018; **33**: 21–35.](https://www.zotero.org/google-docs/?MlHSAz)

[18](https://www.zotero.org/google-docs/?MlHSAz)	[Laurie S *et al.* Genomic reanalysis of a pan-European rare-disease resource yields new diagnoses. *Nat Med* 2025; **31**: 478–489.](https://www.zotero.org/google-docs/?MlHSAz)

[19](https://www.zotero.org/google-docs/?MlHSAz)	[Majeed S *et al.* International policies guiding the selection, analysis, and clinical management of secondary findings from genomic sequencing: A systematic review. *Am J Hum Genet* 2024; **111**: 2079–2093.](https://www.zotero.org/google-docs/?MlHSAz)

[20](https://www.zotero.org/google-docs/?MlHSAz)	[Vears DF, Sénécal K, Borry P. Reporting practices for variants of uncertain significance from next generation sequencing technologies. *Eur J Med Genet* 2017; **60**: 553–558.](https://www.zotero.org/google-docs/?MlHSAz)

[21](https://www.zotero.org/google-docs/?MlHSAz)	[Brown CG *et al.* Detecting the Difficult: An Intronic NPC1 Variant Hiding in Plain Sight. *Am J Med Genet A* 2025; **197**: e64012.](https://www.zotero.org/google-docs/?MlHSAz)

[22](https://www.zotero.org/google-docs/?MlHSAz)	[Pineda M, Walterfang M, Patterson MC. Miglustat in Niemann-Pick disease type C patients: a review. *Orphanet J Rare Dis* 2018; **13**: 140.](https://www.zotero.org/google-docs/?MlHSAz)
